# The Antigen Processing and Presentation Machinery in Lymphatic Endothelial Cells

**DOI:** 10.3389/fimmu.2019.01033

**Published:** 2019-05-07

**Authors:** Laura Santambrogio, Stella J. Berendam, Victor H. Engelhard

**Affiliations:** ^1^Department of Pathology, Microbiology and Immunology, Albert Einstein College of Medicine, New York, NY, United States; ^2^Department of Microbiology, Immunology and Cancer Biology, Carter Immunology Center, University of Virginia School of Medicine, Charlottesville, VA, United States

**Keywords:** lymphatic endothelial cells, MHC class I, MHC class II, antigen processing and presentation, lymph

## Abstract

Until a few years ago, lymphatic vessels and lymphatic endothelial cells (LEC) were viewed as part of a passive conduit for lymph and immune cells to reach lymph nodes (LN). However, recent work has shown that LEC are active immunological players whose interaction with dendritic cells and T cells is of important immunomodulatory relevance. While the immunological interaction between LEC and other immune cells has taken a center stage, molecular analysis of LEC antigen processing and presentation machinery is still lagging. Herein we review the current knowledge of LEC MHC I and MHC II antigen processing and presentation pathways, Including the role of LEC in antigen phagocytosis, classical, and non-classical MHC II presentation, proteasome processing and MHC I presentation, and cross-presentation. The ultimate goal is to provide an overview of the LEC antigen processing and presentation machinery that constitutes the molecular basis for their role in MHC I and MHC II-restricted immune responses.

## MHC I and MHC II Antigen Processing Machinery

### MHC I and MHC II Molecules

Under basal physiological conditions both human and murine lymphatic endothelial cells (LEC) express both MHC class I and MHC class II molecules (1). However, as previously reported for blood endothelial cells (BEC) (2) the level of MHC II expression differs according to the anatomical location from which the cells are isolated (1, 3). LEC from LN (LN-LEC) express a high number of MHC II molecules while LEC from diaphragm express a much lower number (1). The MHC II surface expression in LN-LEC is similar to what observed in BEC but less than fibroblastic reticular cells from LN (1). LEC MHC II molecules are both endogenously synthesized or acquired from hematopoietic cells, as determined by chimera experiments in MHC II^−/−^ mice (1, 4, 5). At the transcription level, MHC II expression is regulated by CIITA, which is not a DNA binding factor but instead a transactivator that regulates quantitative aspects of MHC-II expression by binding the MHC-II enhanceosome (6). CIITA expression is under the control of 4 different promoters (I, II, III, IV) and, in non-professional APC, MHC II expression is mostly regulated by CIITA IV (6). This promoter is responsive to IFNγ and other pro-inflammatory cytokines, which induce MHC II expression/up-regulation in fibroblasts and BEC (6). Similarly, in LEC it has been shown that endogenous MHC II expression is controlled by CIITA IV (4, 5). However, it is interesting to notice that, in contrast to other non-professional APC where pro-inflammatory stimuli greatly up-regulate surface MHC II molecules, pro-inflammatory stimuli induce less robust MHC II up-regulation in LEC (3, 5, 7). In the future, it would be of interest to analyze why, even though LEC express the type IV INFγ-inducible CIITA, they do not strongly up-regulate MHCII during pro-inflammatory conditions (5).

### The Proteasome and TAP

Every cell expresses the constitutive 26S proteasome (8). This large barrel-shaped protein complex is formed in part by the catalytic 20S core, which consists of two pairs of outer α rings organized in seven α (α1–α7) subunits and two pairs of inner β rings organized in seven β subunits (β1–β7). The outer α subunits function as docking domains that regulate access of substrates to the catalytic chamber. Three of the β subunits (β1, β2, and β5) have proteolytic activities, including caspase-like activity (β1), trypsin-like activity (β2), and chymotrypsin-like activity (β5) (9). In the 26S proteasome, this 20S core is capped at both ends by the 19S regulatory complex (9). Ubiquitinated proteins are recognized by the 19S regulatory elements, which transfer them to the 20S for proteolysis (10). Peptides will then be transported in the ER by the transporter associated with antigen processing (TAP) and trimmed by the ER aminopeptidase I (ERAPI). In the ER the MHC class I heavy chain and β_2_m will transiently associates with TAP to load the peptides into the binding groove (11).

Following IFNγ or TNFα stimulation, new proteasome subunits are incorporated to generate the immunoproteasome (4, 12). β1 is exchanged with the large multifunctional peptidase 2 (LMP2) (also known as iβ1 or psmb9). β2 is exchanged with the multicatalytic endopeptidase complex-like-1 (MECL-1) (also known as iβ2 or psmb10). β5 is exchanged with the large multifunctional peptidase 7 (LMP7) (also known as iβ5 or psmb8). The 19S regulatory complex is exchanged with the Proteasome Activator α (PA28α) and PA28β, known as 11S regulator (2). The proteolytic functions of the immunoproteasome are greatly enhanced compared to those of the constitutive proteasome, as the immunoproteasome is more efficient in degrading ubiquitinated proteins and viral proteins, and in generating peptides for MHC-I presentation (13).

Until a few years ago, the presence of the proteasome in LEC was only indirectly analyzed by determining that TAP deficient mice were much less efficient in presenting MHC-I restricted OVA-derived peptides (7). However, a recent paper reported proteasome transcripts in LEC and BEC from different anatomical locations [Table 1 and (14)]. All of these endothelial populations expressed comparable transcript levels for constitutive proteasome subunits and 19S regulatory subunits. However, LEC and BEC from LN expressed 5-8-fold higher levels of transcripts for psmb8, psmb9, and psmb10, and twice as much PA28α and β. This suggests that LN-localized LEC preferentially express immunoproteasomes. Similarly, LN-localized LEC and BEC express 2–6 fold higher levels of TAP1 and TAP2, and twice as much ERAP1 and tapasin. Although these cells were isolated from resting LN, this suggests that their MHC-I processing and presentation capability is elevated.

**Table 1:** Comparative transcriptome profiling of antigen processing and presentation pathway genes from mouse lymphatic and blood endothelial cells.

**Gene**	**Description**	**Lymph node LEC**	**Lymph node blood EC**	**Diaphragm LEC**
**MHC-I AND RELATED**				
B2m	Beta-2 microglobulin	477796^a^	450780	69620
H2-K1	Histocompatibility 2, K1, K region	221754	201711	28827
H2-D1	Histocompatibility 2, D region locus 1	107074	86751	24764
H2-T23	Histocompatibility 2, T region locus 23	10686	9464	6609
H2-M3	Histocompatibility 2, M region locus 3	2375	1656	1029
H2-K2	Histocompatibility 2, K region locus 2	1770	1203	904
Mr1	Major histocompatibility complex, class I-related	1430	722	1134
H2-Ke6	H2-K region expressed gene 6	1373	1642	1671
H2-T10	Histocompatibility 2, T region locus 10	949	635	166
H2-T24	Histocompatibility 2, T region locus 24	771	591	244
Cd1d1	CD1d1 antigen	639	386	197
H2-Q4	Histocompatibility 2, Q region locus 4	443	447	62
H2-Q6	Histocompatibility 2, Q region locus 6	436	347	3
H2-Ke2	H2-K region expressed gene 2	258	321	258
H2-Q8	NA	194	206	4
H2-T3	Histocompatibility 2, T region locus 3	21	2	11
Cd1d2	CD1d2 antigen	20	25	3
H2-M2	Histocompatibility 2, M region locus 2	19	194	4
H2-M5	Histocompatibility 2, M region locus 5	7	14	6
H2-Q1	histocompatibility 2, Q region locus 1	3	3	1
H2-Q10	Histocompatibility 2, Q region locus 10	2	11	6
H2-Bl	Histocompatibility 2, blastocyst	1	2	1
**MHC-II AND RELATED**				
Cd74	CD74 antigen (invariant chain)	1999	2517	64
H2-Ab1	Histocompatibility 2, class II antigen A, beta 1	1063	2305	187
H2-Aa	Histocompatibility 2, class II antigen A, alpha	380	1584	100
H2-Eb1	Histocompatibility 2, class II antigen E beta	304	762	79
H2-DMb1	Histocompatibility 2, class II, locus Mb1	103	85	5
Ciita	Class II transactivator	36	92	13
H2-Ob	Histocompatibility 2, O region beta locus	33	494	8
H2-DMa	Histocompatibility 2, class II, locus DMa	33	83	10
H2-Oa	Histocompatibility 2, O region alpha locus	11	36	0
H2-DMb2	Histocompatibility 2, class II, locus Mb2	9	51	1
H2-Eb2	Histocompatibility 2, class II antigen E beta2	2	26	0
**PROTEASOME**				
Psma1	Proteasome subunit, alpha 1	1804	1990	2194
Psma2	Proteasome subunit, alpha 2	2436	2472	2091
Psma3	Proteasome subunit, alpha 3	1313	1320	1313
Psma4	Proteasome subunit, alpha 4	2137	1939	1765
Psma5	Proteasome subunit, alpha 5	690	680	635
Psma6	Proteasome subunit, alpha 6	4479	4501	4096
Psma7	Proteasome subunit, alpha 7	3964	3890	3738
Psma8	Proteasome subunit, alpha 8	11	52	16
Psmb1	Proteasome subunit, beta 1	3600	3617	3368
Psmb2	Proteasome subunit, beta 2	3303	2471	2984
Psmb3	Proteasome subunit, beta 3	1751	2020	2107
Psmb4	Proteasome subunit, beta 4	2504	2775	2663
Psmb5	Proteasome subunit, beta 5	1405	1376	1159
Psmb6	Proteasome subunit, beta 6	2893	2753	2380
Psmb7	Proteasome subunit, beta 7	4851	5900	2203
Psmb8	Proteasome subunit, beta 8 (LMP7)	4679	4348	564
Psmb9	Proteasome subunit, beta 9 (LMP2)	4288	4159	563
Psmb10	Proteasome subunit, beta 10	5571	6015	1179
Psmb11	Proteasome subunit, beta 11	1	6	3
Psmc1	Proteasome 26S subunit, ATPase 1	1714	1902	1824
Psmc2	Proteasome 26S subunit, ATPase 2	2290	2252	2852
Psmc3	Proteasome 26S subunit, ATPase 3	2431	2573	2450
Psmc3ip	Proteasome 26S subunit, ATPase 3, interacting protein	41	30	58
Psmc4	Proteasome 26S subunit, ATPase, 4	2271	2575	2753
Psmc5	Protease 26S subunit, ATPase 5	1763	1666	1815
Psmc6	Proteasome 26S subunit, ATPase, 6	2164	2496	2319
Psmd1	Proteasome 26S subunit, non-ATPase, 1	2038	2012	2786
Psmd10	Proteasome 26S subunit, non-ATPase, 10	982	698	499
Psmd11	Proteasome 26S subunit, non-ATPase, 11	482	502	450
Psmd12	Proteasome 26S subunit, non-ATPase, 12	2264	2639	2378
Psmd13	Proteasome 26S subunit, non-ATPase, 13	267	272	258
Psmd14	Proteasome 26S subunit, non-ATPase, 14	1289	1302	1376
Psmd2	Proteasome 26S subunit, non-ATPase, 2	2695	2767	3100
Psmd3	Proteasome 26S subunit, non-ATPase, 3	1064	1201	1117
Psmd4	Proteasome 26S subunit, non-ATPase, 4	1057	1213	1307
Psmd5	Proteasome 26S subunit, non-ATPase, 5	726	827	896
Psmd6	Proteasome 26S subunit, non-ATPase, 6	2893	2460	3000
Psmd7	Proteasome 26S subunit, non-ATPase, 7	2678	2492	2579
Psmd8	Proteasome 26S subunit, non-ATPase, 8	2601	2684	2594
Psmd9	Proteasome 26S subunit, non-ATPase, 9	1228	916	1206
Psme1	Proteasome activator subunit 1 (PA28 alpha)	4330	4983	2175
Psme2	Proteasome activator subunit 2 (PA28 beta)	769	903	391
Psme3	Proteasome activator subunit 3 (PA28 gamma, Ki)	2495	2173	1951
Psme4	Proteasome activator subunit 4	2923	2697	2175
Psmf1	Proteasome inhibitor subunit 1	950	918	926
Psmg1	Proteasome assembly chaperone 1	442	414	384
Psmg2	Proteasome assembly chaperone 2	1435	1081	1677
Psmg3	Proteasome assembly chaperone 3	283	232	243
Psmg4	Proteasome assembly chaperone 4	667	591	494
**OTHER PEPTIDASES FOR MHC-I PROCESSING**				
Tpp1	Tripeptidyl peptidase I	11374	9235	6824
Tpp2	Tripeptidyl peptidase II	2910	3005	2429
Nrd1	Nardilysin	2694	2582	3040
Thop1	Thimet oligopeptidase 1	62	66	82
**TAP, TAPASIN, AND ERAP1**				
Tapbp	TAP binding protein	24961	29085	11373
Tap1	Transporter 1, ATP-binding cassette, sub-family B (MDR/TAP)	3796	3489	565
Tap2	Transporter 2, ATP-binding cassette, sub-family B (MDR/TAP)	2398	2223	630
Erap1	Endoplasmic reticulum aminopeptidase 1	1421	1467	655
Tapbpl	TAP binding protein-like	502	589	222
**CATHEPSINS**				
Ctsd	Cathepsin D	32104	8139	14906
Ctsb	Cathepsin B	15143	10660	39555
Ctsl	Cathepsin L	13726	7431	2687
Ctsh	Cathepsin H	3836	1095	1823
Ctss	Cathepsin S	3670	1013	46
Ctsz	Cathepsin Z	3352	3482	2129
Ctso	Cathepsin O	3005	3189	2627
Ctsa	Cathepsin A	2141	2659	1973
Ctsf	Cathepsin F	709	351	790
Ctsk	Cathepsin K	299	60	123
Ctsc	Cathepsin C	89	338	24
Ctsg	Cathepsin G	46	147	2
Ctsw	Cathepsin W	19	53	11
Ctse	Cathepsin E	2	7	0
**CYSTATINS**				
Cst3	Cystatin C	13094	28792	25352
Cstb	Cystatin B	9445	3702	3390
Cst10	Cystatin 10 (chondrocytes)	6582	16279	22
Cst6	Cystatin E/M	67	66	90
Cstad	CSA-conditional, T cell activation-dependent protein	34	74	12
Csta	Cystatin A	11	7	5
Cst7	Cystatin F (leukocystatin)	5	19	2
Cst9	Cystatin 9	0	0	7

a *Data are reported as normalized gene expression levels as Fragments Per Kilobase of transcript per Million mapped reads (FPKM). Data is from Berendam. (14)*.

Other non-proteasomal proteases have been implicated in MHC-I presentation (15). These additional peptidases can trim the proteasome-generated N-extended precursors or even destroy epitopes, by trimming below the size needed for presentation. Among these, LEC from both LN and lymphatic vessels express significant and comparable transcript levels of tripeptidyl peptidases I and II and nardilysin, but negligible levels of thimet [Table 1 and (14)]. The functional implications of these additional LEC proteases, in generating the LEC MHC immunopeptidome, are currently unknown.

### Endosomes and Lysosomes

Late endosomes (LE) and lysosomes (Lyso) are sub-cellular compartments, present in all cell types, specialized for the degradation of endogenous and exogenous materials for maintenance of cellular proteostasis and, in immune cells, for immunosurveillance (16). These organelles characteristically exhibit a low acidic pH, high concentrations of proteases, and expression of lysosome-associated membrane protein (Lamp) protein family members (16). In professional antigen presenting cells, LE and Lyso are also enriched in MHC class II proteins and molecules that regulate peptide loading (Invariant Chain, DM and DO) (17–21) and are referred as MHC class II compartments (MIIC) (22). Ultrastructurally these compartments can appear with different morphologies: multivesicular, multilamellar, or a combination of both (16).

Multivesicular bodies (MVB) are late endosomal compartments with a diameter of between 400 and 500 nm and a limiting membrane that encloses several internal vesicles with diameters of between 40 and 90 nm (16). MVB receive bio-synthetic cargo from the trans-Golgi, cytosolic cargo through autophagy, and exogenous proteins through phagocytosis. MVB are ubiquitously distributed and ultrastructural analysis has shown their presence in LEC (LS, unpublished observation) (23). However, it is currently unknown whether all/or a fraction of these compartments are MHC-II positive and whether there are differences in MHC-II expression in MVBs under steady state and inflammatory conditions. On the other hand the multilamellar bodies (MLB), which are lysosomal-like compartment formed by concentric lamellae and particularly enriched in MHC class II molecules (16) are more specifically expressed in professional APCs, such as DCs, B cells and macrophages, and they have not been found in LEC (LS unpublished observation).

### Invariant Chain, DM, and DO

The MHC II molecules in association with their chaperone Invariant Chain, traffic from the trans-Golgi network to the plasma membrane before internalization to the endosomal MIIC. Sorting signals on the cytosolic tail of the Invariant chain are recognized by the clathrin-coated vesicle machinery for transport to LE/Lyso, where the Invariant chain will be processed by Cathepsins to generate class II-associated invariant chain peptides (CLIP), which occupy the MHC II binding groove and will be exchanged with peptides derived from endosomal processing (24). MHC II/Invariant Chain complexes are present at high levels in LEC and confocal microscopy, performed on primary LEC indicates that MHC II is correctly targeted both at the cell surface and in endosomal compartments (1).

HLA-DM (H-2M in mice) is part of the endosomal antigen processing and presentation machinery and aids peptide loading onto MHC II molecules. HLA-DM (DM) was originally discovered following the analysis of B-cell lines that were inefficient at presenting peptides derived from the processing of phagocytosed proteins but easily presented peptides supplied exogenously (17–19, 25, 26). It was later determined that these cells were defective in the expression of either the HLA-DMA or HLA-DMB genes. Subsequent *in vitro* and *in vivo* experiments determined that the role of DM is to catalyze CLIP removal, stabilize empty MHC II molecules for peptide loading and skew the immunopeptidome repertoire toward high affinity peptides (17, 25, 26). Mice lacking H-2M expressed similar I-A^b^ MHC II cell surface levels as wild type mice. However, the I-A^b^ MHC II molecules were less compact/SDS-resistant and were predominantly associated with CLIP (27). In contrast, lack of DM led to decreased peptide capture by I-A^d^ molecules, but enhanced peptide loading by I-E^d^ MHC II molecules (28). Finally, lack of DM generated a substantial pool of empty or loosely occupied I-A^k^ MHC II conformers with increase peptide binding activity. Mass spectrometry profiles confirmed the presence of an MHC II-peptidome in absence of DM (28, 29). Additionally DM requirements are different for CLIP binding in different registers (30). These results demonstrate that DM has distinct roles depending on its specific class II partners.

Subsequently, an additional protein, DO, was discovered, whose role is to inhibit DM function (18, 31). Importantly, while DM expression is not greatly increased following pro-inflammatory stimuli (TLR activation) that induces dendritic cell maturation, DO is down-regulated (32). As such it was hypothesized that high DO expression in immature dendritic cells would inhibit DM activity and skew the MHC II peptidome toward a broader and less stably bound repertoire. Upon DC maturation, reduced DO expression would lead to high DM activity, shaping the peptide repertoire toward long-lived surface class II MHC complexes, thus promoting productive immune responses (18, 33, 34).

Transcript analysis has shown that Invariant chain, I-A alpha, and I-A beta are expressed significantly in LEC and BEC from LN, but not LEC from lymphatic vessels, but DM and DO expression is very low to negligible, albeit DM is up-regulated following inflammatory stimuli (1, 4) [Table 1 and (14)]. Because removal of CLIP from I-A^b^ molecules is strongly DM dependent, this could explain the inefficient processing and presentation of I-A^b^ restricted antigens by LEC (1). However, the haplotype variation data described above indicate that general conclusions about the ability of LEC to present MHC II restricted antigens should await analysis of other mouse haplotypes.

### Cathepsins

Cathepsins are a large family of serine, cysteine or aspartyl proteases that are present in endo-lysosomal compartments, and may be secreted at steady state or during pathological conditions (35). Cathepsins are most active at acid pH, can still function at neutral pH but are inactive at alkaline pH (36). Although these enzymes are present in most cells, certain cathepsins are enriched in particular antigen presenting cells. For example, Cathepsin S is highly expressed in dendritic cells and B cells, Cathepsin F in macrophages, and Cathepsin L in thymocytes (36–41).

Transcriptome analysis indicated that LEC from vessels express relatively low levels of cathepsins L and F, and negligible levels of Cathepsin S, while the levels of Cathepsin S and L were significantly elevated in LEC from LN [Table 1 and (14)]. However, measured Cathepsin L activity was variable among LN LEC and not evident in LEC from diaphragm (1). The activity of Cathepsin L indicates that at least some LEC could potentially cleave the Invariant Chain and generate CLIP peptides (1). Additionally, LEC could not efficiently process HA (an influenza membrane protein) and the IE-α protein as determined by either CD4 T cell recognition of the MHC II presented HA epitope or FACS analysis using the Y-Ae Ab that recognize I-Ab molecules loaded with the IE-α epitope, either under basal conditions or upon IFNγ stimulation (1). Furthermore, new evidence indicates that LEC express high levels of Cystatin C, B and 10 [Table 1 and (14)], which function as natural inhibitors of cathepsins (42). Altogether, the data point to the possibility that CatL and S activity in LEC is diminished, which could affect the generation of LIP10 and CLIP, and might also diminish the processing of other endogenous antigens.

### Exogenous Peptides Binding and Antigen Exchange

The MHC I and MHC II presented immunopeptidome not only derives from endosomally processed proteins but also from pre-processed peptides that can be directly acquired from the extracellular milieu. Recent proteomic analyses have indicated that processed peptides are present in every biological fluid, among which lymph and blood, have been best characterized (43–50). The Eisen and Raghavan groups demonstrated binding of extracellular peptides to MHC I molecules and their regulation of CD8 T cell function (51, 52). Our group, among others, characterized extracellular peptide binding to MHC II surface molecules (44, 53–57). We determined that peptides carried in lymph were present in the HLA-DR1 immunopeptidome of immature dendritic cells and some of these peptides were not generated by endosomal processing, pinpointing the physiological relevance of MHC II surface/early endosomes loading (44). As such, the peptides present in the lymph, which derive from the metabolic and catabolic process of different parenchymal organs could contribute to the LEC MHC II immunopeptidome, since it has already been shown that LEC can readily bind and present pre-processed peptides (1).

## Phagocytosis and Autophagy

Only very recently LEC have been analyzed for their ability to capture exogenous and endogenous antigens through phagocytosis. *In vivo* experiments using fluorescently labeled OVA indicated that within 90 min the subcutaneously injected protein was identified in LYVE-1^+^ cells, present in LN sub-capsular sinuses (7). Additionally, genes encoding several scavenger receptors, known to be involved in receptor-mediated endocytosis, are upregulated in LEC from lymph node (14). LEC efficiency in processing phagocytosed proteins through the MHC II pathway in steady state condition is low (1); nevertheless LEC can transfer Ags to dendritic cells, which are known to be present in close proximity with LEC in the lymphatic capillary and collectors, to induce CD4 T-cell anergy (1, 58). In addition, LEC efficiently present MHC-I peptides, and it has been reported that phagocytosis in early endosomes can route exogenous antigens (both self and non self) for cross-presentation on MHC class I in a proteasome and TAP-1-dependent manner (1, 3, 7, 58–61). It is interesting to consider that the acquisition of cross-presented material is mediated by these scavenger receptors. A second mechanism that can transfer endogenous proteins in the endosomes is autophagy. Although autophagy has been extensively characterized in BEC (62), there are no reports on the role of autophagy in antigen processing and presentation in LEC.

## LEC and Pathogen Immunity

A growing body of evidences indicates that LEC are involved in immune response to pathogens. It has been recently reported that in extrapulmonary tuberculosis, the lymphatic system is the most common site of infection and LEC function as a niche for *Mycobacterium tuberculosis* (59). Indeed *M. tuberculosis* can replicate in the LEC cytosol and within autophagosomes suggesting that LEC are a previously unrecognized site for infection persistence. Similarly, Hantaviruses have been shown to have a tropism for lymphatic vessels and LEC infection with either Andes virus and Hantaan virus induces LEC hyperpermeabilization and pulmonary edema (63). The edema can be inhibited by α_v_β_3_ integrin as well as VEGFR3 antibodies (63). A LEC role in HIV infection was also reported in promoting infection and latency formation in resting CD4+ T cells (64, 65). Recently an interesting role of LEC in antigen persistence, after resolution of the infection, has been shown (66). After viral challenge and vaccination, the antigen was captured by LEC under proliferative conditions and stored for extended periods of time. This “antigen archiving” mechanism positively influenced the degree of protective immunity provided by circulating memory CD8^+^ T-cells (66, 67).

## Costimulatory and Co-Inhibitory Molecules

Effective activation of T-cells requires the display of MHC-I and MHC-II-peptide complexes as well as an antigen-independent signals provided by co-stimulatory molecules, among which CD40, CD80 (B7.1), and CD86 (B7.2) have been extensively analyzed in their requirements for naïve and memory T-cells activation (68, 69). LN LEC were shown to express very low levels of CD40 and negligible levels of CD80 and CD86 (3, 60). More recent transcriptome analysis has validated these observations, and extended them to include additional costimulatory molecules [Table 1 and (14)]. Importantly these costimulatory molecules did not up-regulate following stimulation with an MHC-I cognate ligand as well as inflammatory signals (TLRs binders or IFNγ) (3, 60).

In contrast, LEC in LN, but not in peripheral tissue lymphatics, express multiple inhibitory receptors that engage counter-receptors on activated T-cells to dampen the immune response (69). These include PD-L1 (CD274) and PD-L2 (CD273), which are present on resting LEC and greatly up-regulated by inflammatory stimuli (1, 70). Interestingly, the ligand for LAG-3, another inhibitory receptor on T-cells, is MHC-II, and induction of CD8 T-cell tolerance by LEC depends on engagement of LAG-3 as well as PD-1 (1, 60). Consequently, it has been suggested that in the absence of functional Ag presentation, the expression of MHC-II molecules on LEC is concerned with inducing Lag-3 mediated tolerance. While the low expression of costimulatory molecules would suggest that LEC would be unable to activate T-cells, they stimulate profound proliferation of CD8 T-cells *in vivo* and *in vitro*, and after peptide pulsing and CD4 T-cell proliferation *in vitro* (58, 60). However, the expression of the co-inhibitory molecules leads to deletional tolerance of CD8 T-cells due to a failure to sustain upregulation of the IL-2 receptor. Thus, LEC represent an important mechanism for mediation of systemic peripheral tolerance (58, 60, 61).

## Exosomes and Other Vesicles

Most cells in the human body release vesicles of different sizes and content which can be classified as apoptotic bodies, micro and macrovesicles and exosomes (71). Exosomes are small (30–120 mm) vesicles generated from the multivesicular late endosomes upon fusion with the plasma membrane and release in the extracellular milieu. Exosomes from different sources have been shown to transport a protein cargo as well as mRNAs and microRNAs. Their physiological and pathological relevance has been established in several immune and cancer-related models (72). Although very little is known about LEC-released exosomes, recently it has been shown that LEC release a vesicular fraction, which includes exosomes, following an inflammatory signal (73). The LEC-derived exosomes are reportedly enriched with a motility-promoting protein signature, which act as a cue for the dendritic cells migratory response (73). In particular LEC released vesicles accumulate in the perivascular stroma of small lymphatic vessels, mostly in the presence of inflammatory cytokines and promote directional migration of CX3CR1-expressing cells (73).

## Concluding Remarks

LEC cells are anatomically placed between parenchymal organs and draining lymph nodes, functioning as a conduit for the lymphatic fluid and are known to control DC and T cell migration in and out of the lymph node (74, 75). During the last few years their functionality in antigen processing and presentation and T cell immune responses has emerged. Under steady-state conditions LEC can present self-antigens to induce T cell tolerance either through expression of peripheral tissue antigens (76) or acquisition of extracellular antigens through phagocytosis or by acquisition of pre-loaded MHC II molecules from DC. Under inflammatory conditions LEC also play an immunosuppressive role by decreasing DC maturation (77) and by up-regulating surface PDL1 (76).

However, the advances in understanding the cross-talk between LEC and T cells has not been paralleled by a detailed mechanistic analysis of their antigen processing and presentation machinery. Characterization of LEC immunoproteasomes, endosomal processing compartments, and antigen acquisition from the lymphatic fluid still needs to be investigated. Nevertheless, the work to date points to an emerging picture of the role played by LEC in maintenance of self-tolerance.

## Author Contributions

LS and VE wrote the review. SB contributed the primary data presented in the table.

### Conflict of Interest Statement

The authors declare that the research was conducted in the absence of any commercial or financial relationships that could be construed as a potential conflict of interest.

## Abbreviations

BEC: blood endothelial cells
LEC: lymphatic endothelial cells
LN: lymph node
LN-BEC: lymph node-associated blood endothelial cells
LN-LEC: lymph node-associated lymphatic endothelial cells
DC: dendritic cells
LE: late endosomes
Lyso: lysosomes
MIIC: MHC class II compartments
MVB: multivesicular bodies
MLB: multilamellar bodies
DM: HLA-DM
Ii: invariant chain
DO: HLA-DO.
